# Supplementary material for: The Study of the Caudal Vertebrae of Thick-Toed Geckos after a Prolonged Space Flight by X-ray Phase-Contrast Micro-CT
Source: Cells. 2023 Oct 7;12(19):2415. doi: 10.3390/cells12192415 (PMC10572532; doi:10.3390/cells12192415)
Supplement: Supplementary file 1 [file cells-12-02415-s001.zip › cells-2506423-supplementary.pdf]

Supplementary materials

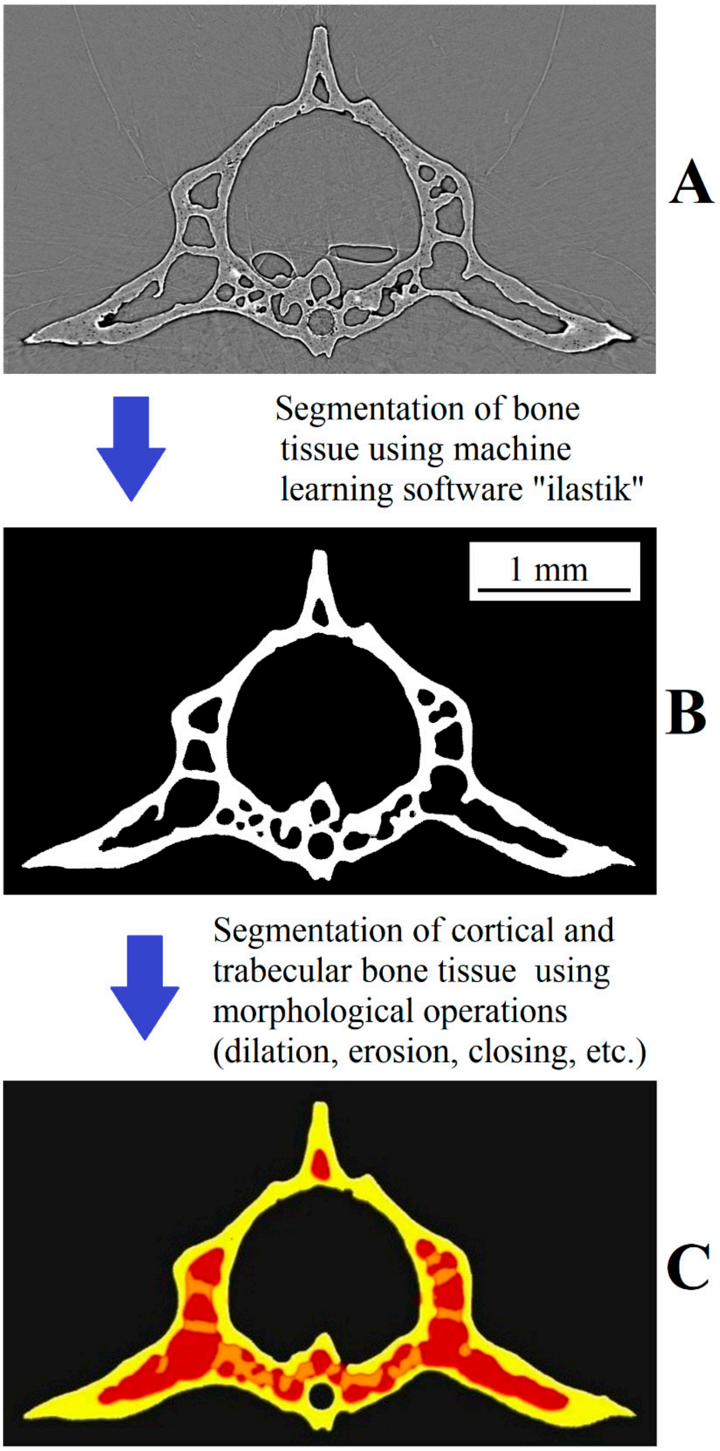

Figure S1. The steps of segmentation of the vertebrae. **A** – XPCT transverse section of the vertebra after reconstruction; **B** – the segmented bone tissue of the vertebra; **C** – the segmented cortical bone (yellow) and the subcortical volume (red) with trabeculae (orange).
